# Tensile response of Ultra High Performance PE Fiber Reinforced Concretes (PE-UHPFRC) under imposed shrinkage deformations

**DOI:** 10.1617/s11527-021-01621-0

**Published:** 2021-05-06

**Authors:** A. Hajiesmaeili, M. A. Hafiz, E. Denarié

**Affiliations:** Maintenance and Safety of Structures, Ecole Polytechnique Fédérale de Lausanne, Station 18, GC A3 398, 1015 Lausanne, Switzerland

**Keywords:** UHPFRC, UHMW-PE, PE-UHPFRC, Autogenous shrinkage, Eigenstresses, TSTM, Tensile response

## Abstract

PE-UHPFRC is a new Ultra High-Performance Fiber Reinforced Concrete (UHPFRC), which is developed to reduce the environmental impact of conventional UHPFRC by replacing the steel fibers with synthetic ones and reducing the clinker content in the mix. The development of the dynamic elastic modulus, the evolution of free autogenous deformations and the eigenstresses development with age, under full and partial restraint conditions, were investigated for PE-UHPFRC and the results were put into perspective with that for conventional UHPFRC with steel fibers. Furthermore, the tensile responses of different mixes under imposed shrinkage were compared and discussed. The results showed a shorter setting time and consequently an earlier initiation of elastic modulus development for PE-UHPFRC compared with that of conventional UHPFRC. Furthermore, the developed eigenstresses under full restraint conditions in a PE-UHPFRC layer compared with that for conventional UHPFRC were reduced by more than 70%, which is highly beneficial especially for cast-in-place rehabilitation applications.

## Introduction

The aging of transportation infrastructures together with the increasing demand of the society have intensified the need for effective and sustainable structural rehabilitation and strengthening techniques. In this regard, the concept of rehabilitation and strengthening of existing bridges with UHPFRC, using a thin UHPFRC overlay in zones of severe mechanical and environmental exposure, has gained the ground over the past decades [1].

Composite UHPFRC-Concrete members are subjected to internal deformations and to external actions. The internal autogenous deformations are mainly due to chemical shrinkage, self-desiccation after setting, drying shrinkage (more or less pronounced depending on UHPFRC recipes) and thermal effects caused by the heat of hydration in the new layer. These deformations starting at early age are restrained by the existing structure as well as the reinforcement bars, if any, in the UHPFRC layer [2]. The very high degree of restraint (load level of the overlay with respect to that for full restraint) of cast-in-place applications with thin overlays (often close to 90%) [3] leads to the development of pronounced tensile eigenstresses in the UHPFRC layer [4–7] which constitute a net loss of the performance in terms of potential tensile capacity.

Several authors reported results on the effect of restrained shrinkage for UHPFRC mixes with steel fibers. Schachinger et al. [8, 9] investigated the cracking risk of two types of UHPC with CEM I 42.5 R/HS and CEM III B 42.5 NW/HS (HS: resistant to sulfates, NW: low heat of hydration), due to restrained autogenous deformation until 28 days, and reported that a cracking risk exists within the first 15 h for the mixes with CEM I 42.5 R/HS. Habel et al. [6] studied the evolution of autogenous deformations and their associated eigenstresses in UHPFRC under incremental restraint, at 20 °C. The comparison of the developed eigenstresses and tensile properties of UHPFRC until 7 days showed that the cracking risk is higher during the first two days. The study of Kamen et al. [4] on the thermo-mechanical response of UHPFRC at early age showed that even though increasing the curing temperature from 20 to 30 °C prepones the initiation of eigenstresses development, it has a negligible effect on the eigenstresses at 7 days and no evidence of damage was observed. Yoo et al. [10] studied the effect of the restraint ratio on the cracking behavior of UHPFRC until 7 days by conducting restrained ring-tests with different ring thicknesses, in which the specimen with thicker steel rings (corresponding to a higher degree of restraint) demonstrated a faster cracking time than the ones with thinner steel rings. The influence of the degree of restraint on the cracking risk was confirmed by [11] who investigated the effect of specimen thickness on restrained shrinkage and cracking behaviors of thin UHPFRC slabs until 9 days. The authors studied two different thicknesses of 35 and 45 mm for the UHPFRC layer and showed that the degree of restraint and the cracking risk were reduced as the thickness of the specimen increased. Furthermore, Weimann et al. [12] reported multiple cracks with crack widths smaller than 50 µm in Engineered Cementitious Composites (ECC), in a ring test subjected to approximately 90% restrained shrinkage, due to the relatively low tensile elastic limit of this material.

Even though it was shown that the cracking risk is higher at an early age for UHPFRC mixes, it should also be considered that eigenstresses continue to increase with age as a consequence of the autogenous shrinkage, and they may reach the Strain hardening domain at a later age. However, only a few notable studies investigated the eigenstresses development in UHPFRC beyond 28 days. Hafiz et al. [13] studied the development of eigenstresses in UHPFRC under full restraint conditions and showed that these stresses could reach values higher than the elastic limit and even reach the tensile strain hardening domain of the material after one month at 20 °C curing.

PE-UHPFRC is a UHPFRC mix in which 50 vol% of the clinker is replaced with limestone fillers [14] and the steel fibers are replaced with Ultra High Molecular Weight Polyethylene (UHMW-PE) ones. This newly developed mix has equivalent or better properties (tensile strain hardening) than mixes realized with steel fibers, adapted for cast-in-place applications of reinforcement of existing structures (sort UA after [15]). Furthermore, it has the potential to reduce the eigenstresses in UHPFRC by reducing the autogenous shrinkage and increasing the viscoelastic response of the material. Kang et al. [16] showed that autogenous shrinkage decreases proportionally with the increase in limestone filler content. The autogenous shrinkage decreased by more than 30% by replacing 50% mass of cement with limestone filler. Furthermore, Hafiz et al. [13] reported a higher viscoelastic response for a mix with 50% mass replacement of clinker with limestone filler. Additionally, Bissonnette et al. [17] found higher tensile creep for a concrete mix with smaller diameter steel fibers due to the influence of the fibers on the microstructure of the paste at the paste fiber interface. Therefore, the much smaller diameter of the PE fibers (0.012 mm) compared with that of steel fibers (0.16 to 0.2 mm) can have a potential to increase the tensile creep of PE-UHPFRC, and consequently reduce the eigenstresses by providing greater stress relaxation compared to the mixes with steel fibers.

The present study reports on the delayed response under imposed shrinkage deformations of PE-UHPFRC compared with that of conventional UHPFRC. In a first step, the investigated materials and their properties are presented. Secondly, the methods are described, highlighting the use of a Temperature Stress Testing Machine (TSTM) to investigate the eigenstresses development under full restraint conditions, and the Vibration Resonance Frequency test (VRF) to study the development of the dynamic elastic modulus with age. The development of the elastic modulus, the evolution of autogenous deformations, and the development of eigenstresses are investigated for three types of UHPFRC mixes including (1) PE-UHPFRC with PE fiber and 50 vol% replacement of clinker with limestone filler, (2) S-UHPFRC with steel fibers, and (3) S-LF-UHPFRC with steel fibers and 50% mass replacement of clinker with limestone filler. Finally, the tensile responses of different UHPFRC mixes under imposed shrinkage deformations are put into perspective and the effect of the viscoelastic response on reducing the apparent elastic modulus of different mixes at very low loading rates is discussed.

## Materials

Four mixes were used: (1) a newly developed UHPFRC mix, named PE-UHPFRC, in which the steel fibers were fully replaced by UHMW-PE ones and 50% volume of the clinker was replaced by limestone fillers [18], (2) a conventional UHPFRC mix with steel fibers from the CEMTEC_multiscale_^©^ family [19], optimized and modified at MCS/EPFL [20, 21], designated as S-UHPFRC, (3) a UHPFRC mix based on S-UHPFRC in which 50% of the cement was replaced with inert limestone filler henceforth referred to as S-LF-UHPFRC [13], and (4) a mix similar to that of PE-UHPFRC with less sand and without quartz powder, named PE-UHPFRC(*). Six different powders including cement CEM I 52.5 HTS Lafarge, two types of limestone fillers of different gradings: Betoflow D® and Betocarb SL® (OMYA), white silica fume from SEPR (BET = 14 m^2^/g), quartz powder (*d*_50_ = 11.4 μm) and fine quartz sand (*d*_50_ = 250 μm) were used in the mixes. The detailed compositions of the mixes are given in Table 1.

**Table 1:** Mix proportions of the PE-UHPFRC, PE-UHPFRC(*), S-UHPFRC and S-LF-UHPFRC

Components (kg/m^3^)	(1) PE-UHPFRC	(2) S-UHPFRC	(3) S-LF-UHPFRC	(4) PE-UHPFRC(*)
Cement	508	1467	733.7	627.8
Silica fume	178	381.4	293.5	220
Betocarb®-HP SL	170	–	223	210
Betoflow®-D	389	–	501.6	480.6
Fine Sand	525	–	–	348.4
Quartz Powder	223	–	–	–
Water	165	225.8	217.9	224
Water/fines	0.124	0.129	0.129	0.146
Water/cement	0.357	0.163	0.310	0.357
UHMW-PE fiber	19.6	–	–	19.6
Steel fibers	–	706.5	706.5	–
HRWRA	27	20.5	14.7	26.4
Ca(NO_3_)_2_	11	–	–	13.2
Defoaming agent	0.1	–	–	0.1

The PE-UHPFRC and PE-UHPFRC(*) have a fibrous mix containing 2 vol% UHMW-PE fiber (*l*_f_ = 6 mm, *d*_f_ = 0.012 mm) type SK99 (*E* modulus = 155 GPa) and SK 62 (*E* modulus = 86 GPa), respectively. The fibrous mix of both S-UHPFRC and S-LF-UHPFRC consisted of two types of micro (with a semi-circular section with variable dimensions) and macro (with *l*_f_ = 10 mm, *d*_f_ = 0.2 mm) fibers with a total dosage of 9 vol%. It should be noted that due the different geometry of the PE and steel fibers, there are almost 100 times (2/9 × 10/6 × 0.2^2^/0.012^2^) more individual fibers in PE mixes compared to that of steel fiber mixes, despite a higher fiber volume (9%) in the latter.

**Fig. 1:**
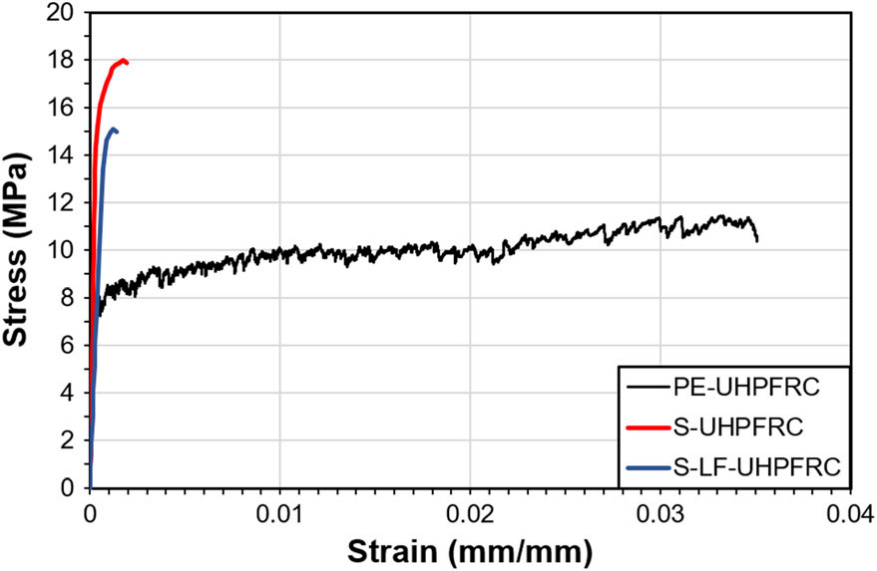
Tensile response of the PE-, S-, and S-LF- UHPFRC mix (for direct comparison, only the strain hardening response of the three mixes is shown)

The strain hardening part of the tensile response of the mixes 1, 2, and 3 are illustrated in Fig. 1. Furthermore, the mechanical properties of the same mixes in the hardened state are shown in Table 2. The tensile properties are the average of 15 and 8 uniaxial tensile tests on dumbbell specimens with a thickness of 30 mm, for PE and steel fiber UHPFRC mixes, respectively. The compressive strength is the average of results on three cylinders after [15]. The average compressive strength of mix PE-UHPFRC(*) on three cylinders 7/14 cm after [15] was 119.7 at 28 days.

**Table 2:** Mechanical properties of the PE-UHPFRC, S-UHPFRC, and S-LF-UHPFRC, average (standard deviation), at 28 days

Properties	PE-UHPFRC	S-UHPFRC	S-LF-UHPFRC
Tensile strength (MPa)	11.7 (0.6)	18 (3.1)	15.1 (2.7)
Tensile elastic limit (MPa)	7.7 (0.5)	12.3 (1.7)	11.1 (1.9)
Tensile strain at peak stress (‰)	35 (10)	1.64 (0.44)	1.27 (0.51)
Compressive strength (MPa)	120 (2.6)	230.5 (0.85)	169.7 (0.5)
Young’s modulus^a^ (GPa)	42.6 (2.2)	51 (2.3)	46.3 (1.3)

^a^After SIA 262/1, Annex G

As shown in Fig. 1, the fiber type highly influences the tensile behavior of UHPFRC mixes, steel fibers lead to higher strength while PE fibers offer higher strain capacity [22]. In addition, it can be seen that replacing 50% of the clinker with limestone filler slightly reduces both tensile strength and tensile elastic limit in steel fiber UHPFRC mixes.

## Experimental procedure

### Delayed response

**Fig. 2:**
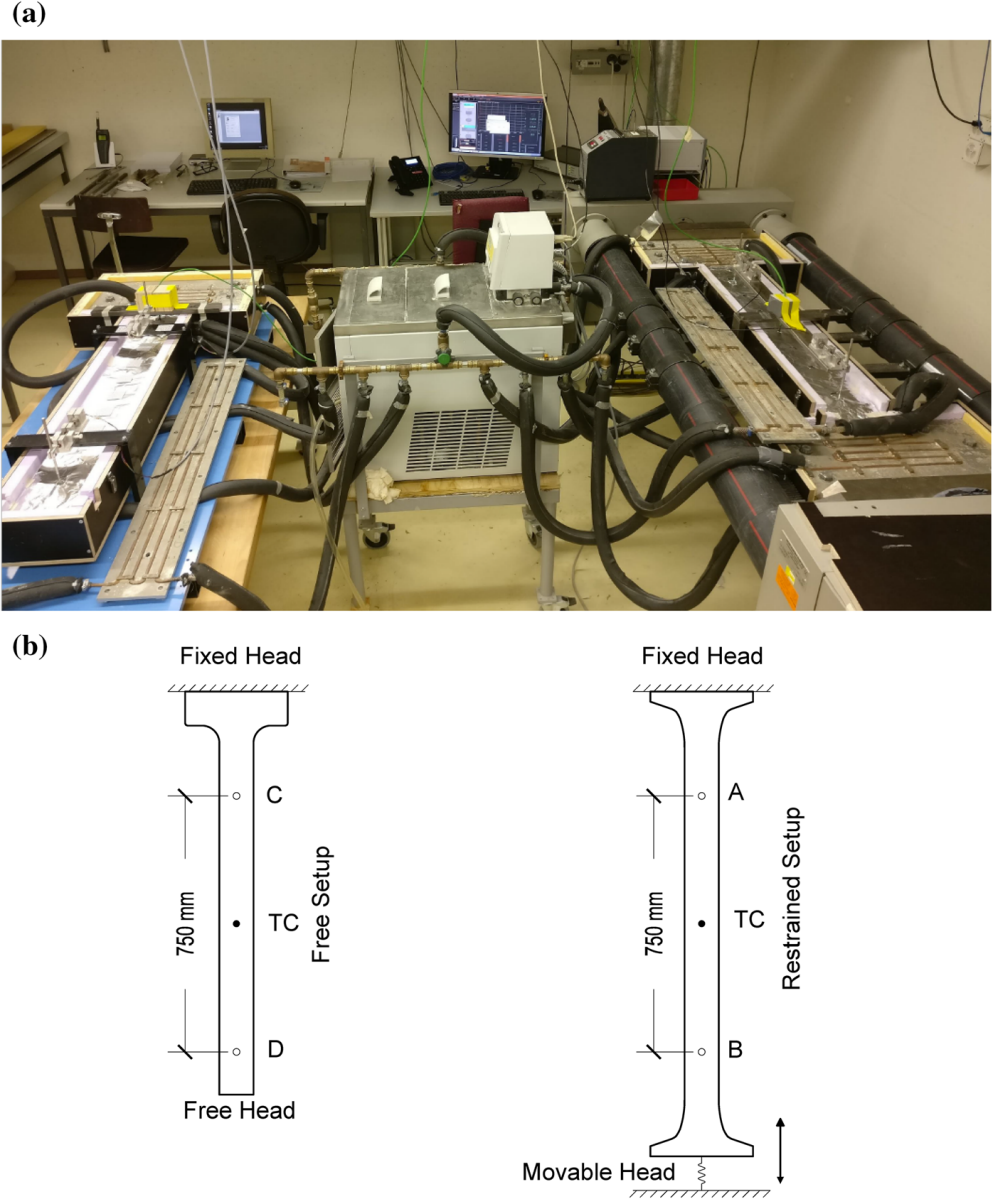
TSTM setup: **a** overview with cooling system, **b** schematic representation of the specimen’s geometry and location of measurement points of transducers (A, B, C, D) and thermocouples (TC)

The free autogenous deformations and eigenstresses development of the UHPFRC mixes under full and partial restraint conditions were investigated using a Temperature Stress Testing Machine [23–25] at MCS/EPFL [26] from a very early age, directly after casting. Quasi–isotherm temperature conditions of 20 °C were ensured in the specimen with the help of a cooling circuit surrounding the molds. The TSTM setup is shown in Fig. 2.

The setup consisted of two parts. The free autogenous deformations were measured with the Free Setup and the eigenstresses development under full and partial restraint conditions were investigated using the Restrained Setup with the help of a load cell and an electromechanical actuator linked to a closed loop control system. The associated free deformations in the restrained specimen, as well as the free deformations in the free system, were determined by means of the measurements of two LVDT in each specimen, attached to a rod inserted in the material immediately after casting, through openings in the molds. In the Restrained Setup, two LVDT (A and B) with a range of ± 0.5 mm were placed 750 mm apart, whereas in the Free Setup, two LVDT (C and D) with a range of ± 2.5 mm were installed 750 mm apart from each other, as shown in Fig. 2. The eigenstresses were calculated by dividing the force measured by the load cell with the cross-sectional area of the specimen. In both devices, fully sealed specimens of cross-sectional dimensions 50 mm × 100 mm were used. Further details regarding the TSTM setup can be found in [26–29].

The autogenous shrinkage was calculated by dividing the difference of displacements shown by LVDT C and D by the distance between them, assuming a linear variation of the autogenous deformation from one end to the other in the Free Setup. The partial restraint test was conducted using stroke control, in which the stroke was kept in the same relative position without any movement, throughout the test. The partial restraint imposed by the finite stiffness of the machine in this test was determined by comparing the free deformations in the restrained system and the free deformations in the free system. For the full restraint tests, in order to ensure fully restrained condition in the Restrained Setup, the deformations were controlled actively after setting to keep the relative displacement between the two points A and B at zero. However, at the fresh state and in the very early age close to setting time, the material stiffness was too low to impose a closed loop force or deformation control. As such, the tests were started under stroke control (passive control) with the stroke remaining in the same position as that at the start of the test. The monitored development of eigenstresses under passive stroke control was used to determine the stress to activate the deformation control leading to full restraint condition. A value of 0.2 MPa in tension (equivalent to 100 kg acting on the specimen cross section) was chosen as the trigger value, to be low enough to minimize the impacts on the viscous effects of the loading history induced by the passive stroke control timeframe after setting, while being high enough to reach a sufficient stiffness of the specimen to respond to a closed loop deformation control without yielding out of control.

Table 3 gives an overview of all the tests performed using the TSTM set-up. For mixes 1, 2, and 3, two full restraint tests were carried out. Additionally, one partial restraint test was carried out on PE-UHPFRC(*).

**Table 3:** Overview of the performed tests with TSTM

Material	Duration	Restraint condition
PE-UHPFRC	30 days and 65 days	Full
S-UHPFRC	31 days and 65 days	Full
S-LF-UHPFRC	32 days and 65 days	Full
PE-UHPFRC(*)	28 days	Partial (0.65)

### Development of elastic modulus

**Fig. 3:**
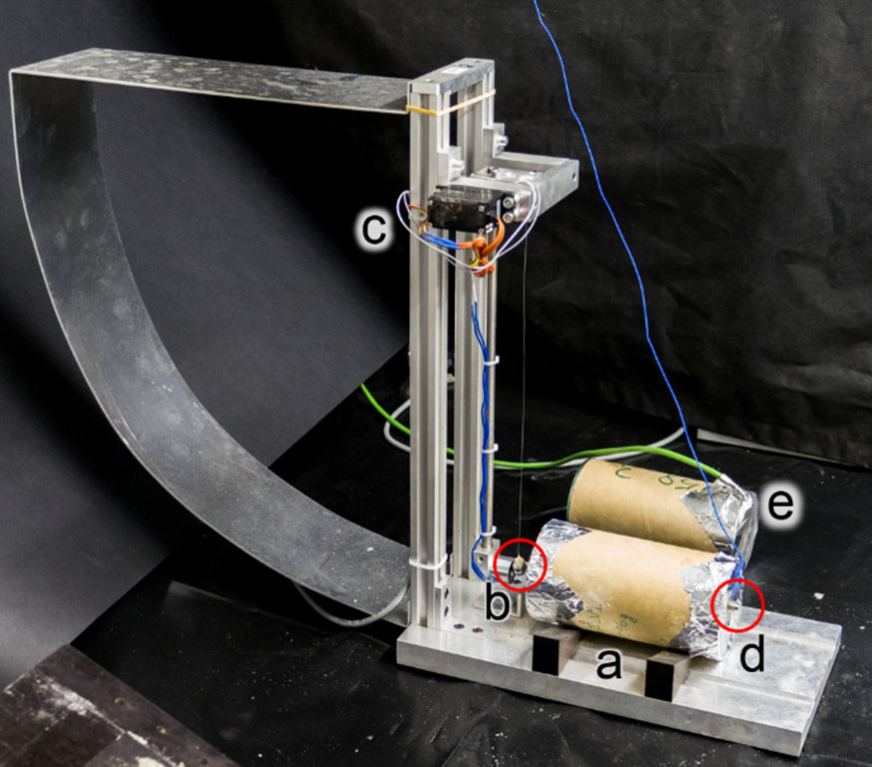
VRF setup: **a** cylindrical VRF specimen, **b** steel ball, **c** automatic hitting system, **d** accelerometer, **e** specimen for measuring temperature evolution

The vibration resonance frequency (VRF) test method [27, 30] was used to determine the dynamic elastic modulus of the mixes from an early age and assess its development with age. The method is based on the measurement and analysis of natural vibration frequencies of the first two longitudinal modes of vibration of a cylindrical specimen using the Rayleigh–Ritz method, which relates the frequencies of vibration to the elastic modulus and the density of the material for a given geometry. Cylindrical specimens with a diameter of 70 mm and a length of 140 mm were used for the tests. The measurements of the frequencies started shortly after setting time of the material. A 10 mm diameter steel ball suspended with a steel thread was used to hit the center of one of the circular faces of the specimen. The hits were done automatically using an arm connected to a step motor with a magnetic head, once per minute. The propagated longitudinal waves were then captured by a miniature accelerometer at the opposite face of the specimen. The captured waves were digitized and analyzed using signal processing to obtain the 1st and 2nd resonance frequency of the specimen. The elastic modulus at different age was calculated using the resonance frequencies of the specimen and the density of the material (Fig. 3). In order to estimate the setting time and start the measurement from the beginning of elastic modulus development, the temperature evolution was monitored in another specimen, using a thermocouple inserted right after casting.

## Results and discussion

### Development of elastic modulus

**Fig. 4:**
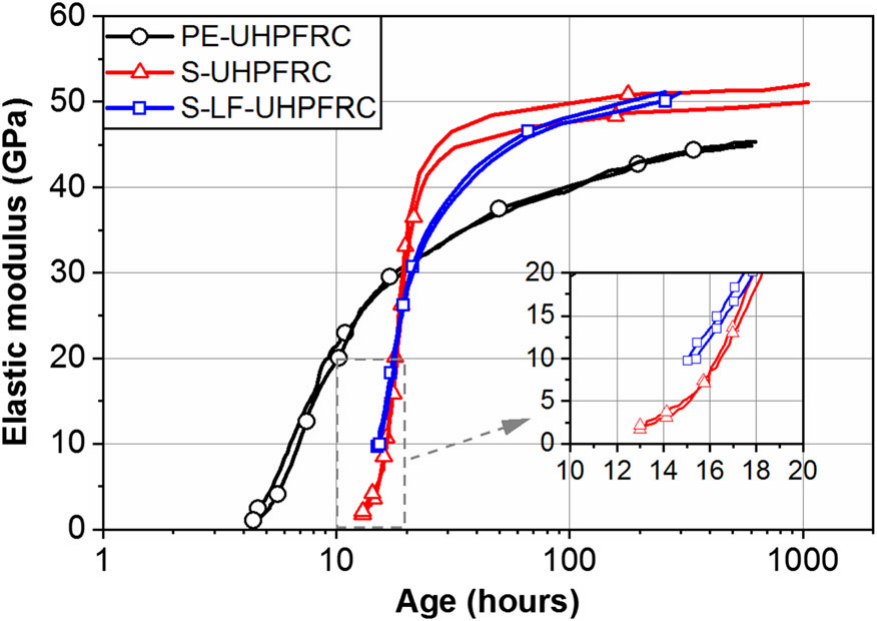
Development of dynamic elastic modulus for PE-UHPFRC, S-UHPFRC, and S-LF-UHPFRC

The development of the dynamic elastic modulus is shown in Fig. 4 on a semi-log scale for the mixes 1, 2 and 3. As expected, the final dynamic elastic modulus of the mix with steel fibers was higher than that for the PE-UHPFRC. The dynamic elastic modulus was slightly higher than the static ones (Table 2) and reached above 51 GPa in S-UHPFRC and S-LF-UHPFRC mixes, and 45 GPa in PE-UHPFRC mixes, after 14 days.

The measurements on the S-LF-UHPFRC specimens started at the age of 15 h, when the dynamic elastic modulus had already reached 10 GPa. However, from the trend of the curves, it can be seen that the development of the elastic modulus started in S-LF-UHPFRC slightly earlier than in S-UHPFRC, and it started in PE-UHPFRC approximately 10 h earlier than the other two mixes. The difference between the results for the two steel fiber mixes can be explained considering the difference in their superplasticizer dosage. S-LF-UHPFRC had 14.7 kg/m^3^ superplasticizer whereas S-UHPFRC had 20.5 kg/m^3^. The retarding effect of the superplasticizer can delay the setting time and consequently the initiation of the elastic modulus development. The considerable difference between the PE-UHPFRC and the other two mixes can be attributed to the accelerating effect of Ca(NO_3_)_2_ used in the mix with PE fibers. Bost et al. [31] showed a significant accelerating effect of calcium nitrate on Portland cement even at a very low concentration. Thus, the Ca(NO_3_)_2_ in PE-UHPRC mixes reduced the setting time and accordingly preponed the initiation of elastic modulus development. Additionally, the effect of limestone filler in accelerating the hydration of cement grains by acting as crystallization nucleus [32], and in reducing the interparticle spacing product (at a constant liquid content and for limestone particles finer than cement) [33] played a role in reducing the setting time and therefore bringing forward the initiation of the elastic modulus development in PE-UHPFRC and S-LF-UHPFRC. A similar trend was reported in [33–35].

**Fig. 5:**
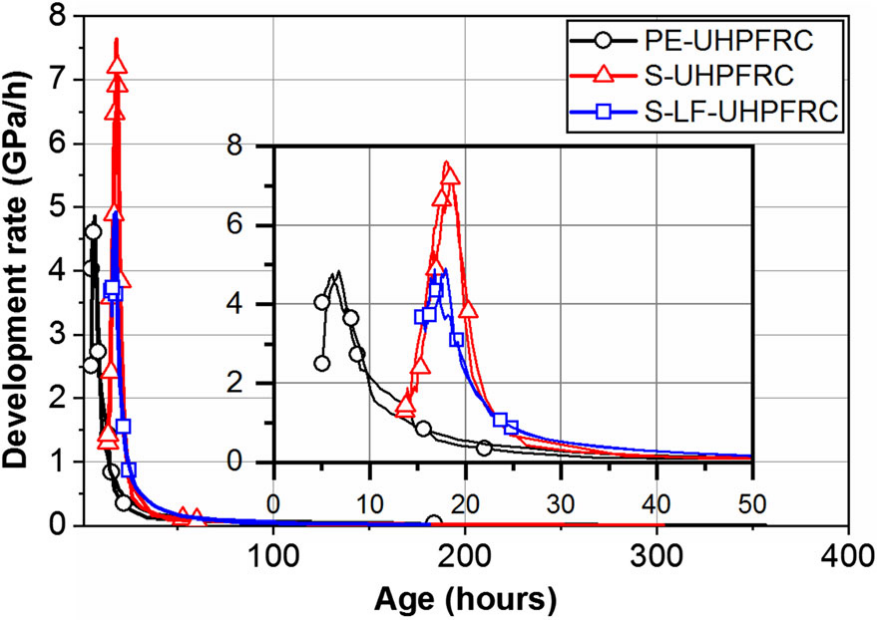
Rate of elastic modulus development in PE-UHPFRC, S-UHPFRC, and S-LF-UHPFRC

Even though the development of the elastic modulus started earlier in the PE-UHPFRC mix compared with that for the other two mixes, at the age of 20 h all the mixes reached an elastic modulus of 30 GPa as the result of different development rates. Figure 5 shows the rate of elastic modulus development in UHPFRC mixes 1, 2, and 3. The rate of elastic modulus development had a higher peak in the S-UHPFRC mix compared with the other two mixes which had a similar trend. This can be attributed to the dilution effect of the limestone filler by increasing the water/cement (*w*/*c*) ratio in the mixes. The *w*/*c* ratio of the S-UHPFRC was 0.15 while the *w*/*c* ratio of PE-UHPFRC and S-LF-UHPFRC were 0.32 and 0.30, respectively. Considering the increase in the rate of drop of relative humidity with a decrease in the *w*/*c* ratio [36] and taking into account the similar kinetics of the rate of hydration and the changes in the relative humidity, the different rates of elastic modulus development of the mixes can be explained. The rate of elastic modulus development approached zero after 14 days.

### Free autogenous deformations

**Fig. 6:**
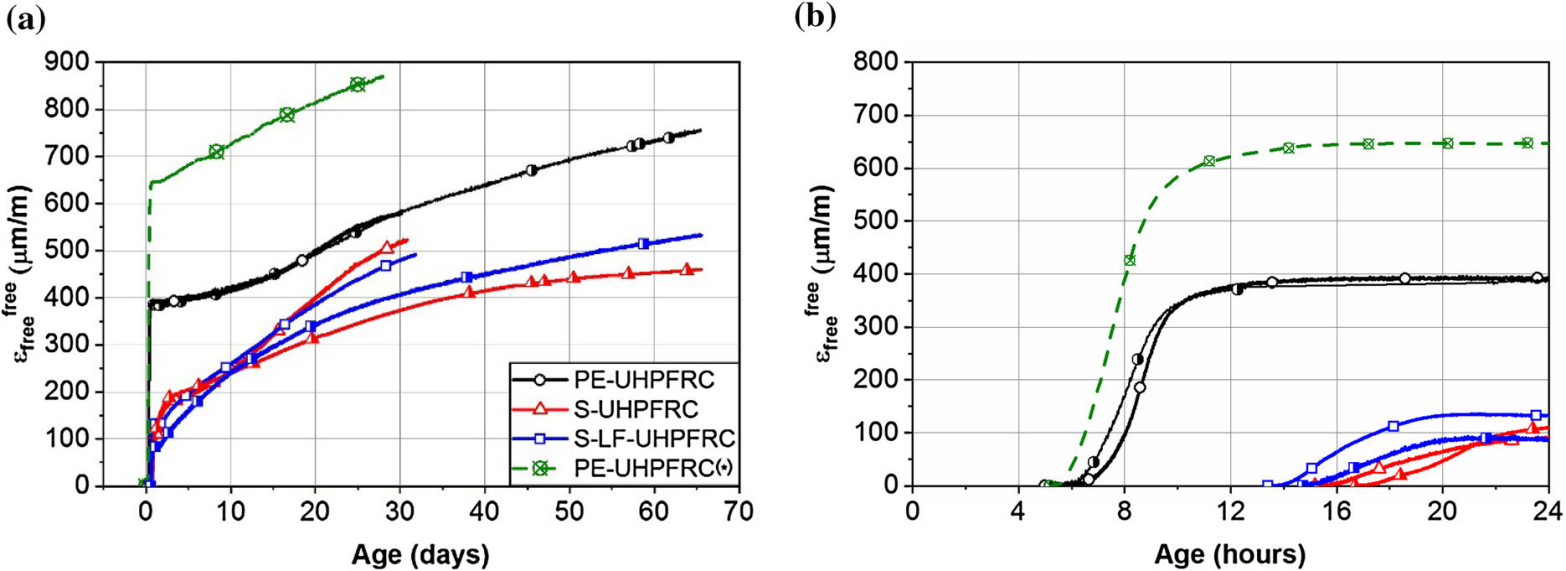
Evolution of free autogenous deformations of different UHPFRC mixes (**a**) zoom until 24 h (**b**) (increasing strains indicate shrinkage)

The evolution of the free autogenous deformations (*ε*_free_^free^) of the UHPFRC mixes are presented in Fig. 6. In this figure, the deformations are zeroed at the end of swelling and increasing strains indicate shrinkage. All the mixes showed a fast autogenous shrinkage in the first few days. This trend can be explained by the progress of the hydration process and refinement of the pore structure with fast consumption of water [27].

The results of both full restraint test series on the PE-UHPFRC mix were almost superimposed which showed a very good reproducibility. The PE-UHPFRC(*) mix showed a higher shrinkage than that in PE-UHPFRC at an early age until 12 h. The higher autogenous shrinkage of PE-UHPFRC(*) can be explained by a lower sand content in this mix (348.5 kg/m^3^ compared with 525 kg/m^3^ in PE-UHPFRC) and also its higher water/fines ratio. Cheyrezy and Behloul [37] found that below w/b of 0.17, the autogenous shrinkage decreases with the w/b ratio. The autogenous deformations were higher in PE-UHPFRC compared to that in UHPFRC mixes with steel fibers. The stiff and very dense steel fibers skeleton in S-UHPFRC and S-LF-UHPFRC resists against the autogenous deformations and reduces the autogenous shrinkage at early ages [38–40] while the flexible PE fibers cannot hinder those autogenous deformations. This difference was reduced as the stiffness of the matrix increased with age and the role of the fibers’ stiffness in controlling the deformations was lessened. A trend similar to that of PE-UHPFRC mixes was reported for the autogenous deformations of a PVA fiber reinforced Engineered Cementitious Composites (ECC) in [41].

Contrary to the finding of [16] regarding the effect of limestone filler in reducing the autogenous shrinkage in UHPFRC mixes, no considerable difference was observed between the autogenous shrinkage of S-UHPFRC and S-LF-UHPFRC in which 50% of cement was replaced with limestone filler. This discrepancy can be explained by considering a similar effect as that of the relative volume fraction of the fibrous mix and sand on the yield stress of the fresh mix, which indicates the resistance of a material against deformation, for autogenous shrinkage. Martinie et al. [42] showed that the yield stress of fresh cementitious composites is strongly correlated to the sum of the relative volume fraction of the fibers and the relative volume fraction of the granular skeleton: *Φ*_f_/(*r*/4) + *Φ*_s_/*Φ*_m_, where *Φ*_f_ is the volume fraction of fibers, *r* is the aspect ratio of the fiber (length/diameter), and *Φ*_s_ and *Φ*_m_ are the volume fraction and the dense packing fraction of the sand respectively. The investigated mix in [16] had 162 kg/m^3^ steel fibers (13/0.2 mm) and 873 kg/m^3^ of fine sand while the S-UHPFRC and S-LF-UHPFRC mixes had 471 kg/m^3^ steel fibers (10/0.2 mm) and 235.5 kg/m^3^ of steel wool that has a similar effect as sand. Considering 0.6 and 0.13 (experimental values) for dense packing fractions of the sand and the steel wool, respectively, the total relative volume fraction of the rigid inclusions is 0.87 and 0.98 for the mix studied in [16] and the UHPFRC mixes with steel fiber in this study, respectively. Thus, similar to the effect of increasing the total relative volume fraction of the rigid inclusions on increasing the yield stress of the fresh mix, it can be assumed that increasing the total relative volume fraction of the rigid inclusions in S-UHPFRC and S-LF-UHPFRC led to increasing the resistance to deformation and reduced the effect of limestone filler on the autogenous shrinkage.

### Eigenstresses under full/partial restraint

**Fig. 7:**
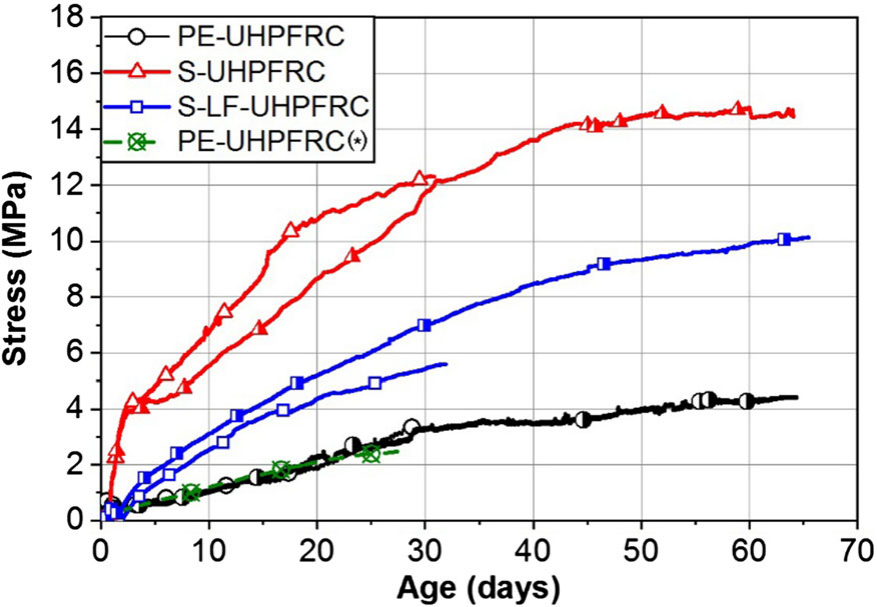
Eigenstresses development of different UHPFRC mixes, quasi isotherm curing conditions at 20 °C

Figure 7 shows the eigenstresses development of PE-UHPFRC, S-UHPFRC, and S-LF-UHPFRC under full restraint and PE-UHPFRC(*) under partial restraint condition. The degree of restraint was 0.65 for the partial restraint test, estimated from the asymptotic ratio after 90 days, of the free deformations in the restrained set-up to the free deformations in the free set-up) per equation (*ε*_restrained_^free^/*ε*_free_^free^). Even though the autogenous shrinkage of PE-UHPFRC(*) was approximately 1.5 times higher than that for the PE-UHPFRC mix, the eigenstresses development of these two mixes followed a very similar trend and were almost superimposed, despite the very different degrees of restraint. Considering similar viscoelastic responses for the two PE fiber mixes, this can be explained by the opposing effects of a higher shrinkage (almost 1.5 times more for mix 4) but of a lower restraint (0.65) also for mix 4 (1.5 × 0. ≈ 1).

The value of eigenstresses in a 50 mm thick layer of PE-UHPFRC at 28 days decreased by a factor of 2 and 4 compared with that for S-LF-UHPFRC and S-UHPFRC, respectively. The lower value of eigenstresses in PE-UHPFRC might be explained in two levels: from a matrix and from a fibrous mix point of view. Regarding the matrix, Hafiz et al. [13] showed a higher viscoelastic response for the mixes with a higher dosage of limestone filler in creep tests starting at 14 days age, which consequently results in a higher relaxation potential and help to mitigate the developed eigenstresses. Concerning the fibrous mix, it was shown that using fibers (steel or synthetic ones) in a cementitious matrix increases creep in tension [17, 43, 44] especially in the case of fibers with a smaller diameter [17]. Thus, the smaller diameter and higher number of the PE fibers compared to the steel fibers can lead to higher tensile creep in PE-UHPFRC mixes. Furthermore, Garas et al. [45] found a higher tensile creep for the UHPFRC specimens cured at room temperature compared with that for the thermally treated specimens due to the relatively lower stiffness of fiber/matrix interfaces in the former specimens. Accordingly, a higher volume of relatively low-stiffness fiber/matrix interface in PE-UHPFRC mixes due to the higher number of PE fibers compared with steel fibers may lead to a higher tensile creep in these mixes. Consequently, the higher tensile creep capacity of PE-UHPFRC mixes can provide greater stress relaxation compared to the mixes with steel fibers and reduce the developed eigenstresses.

### Tensile response under imposed shrinkage deformations

**Fig. 8:**
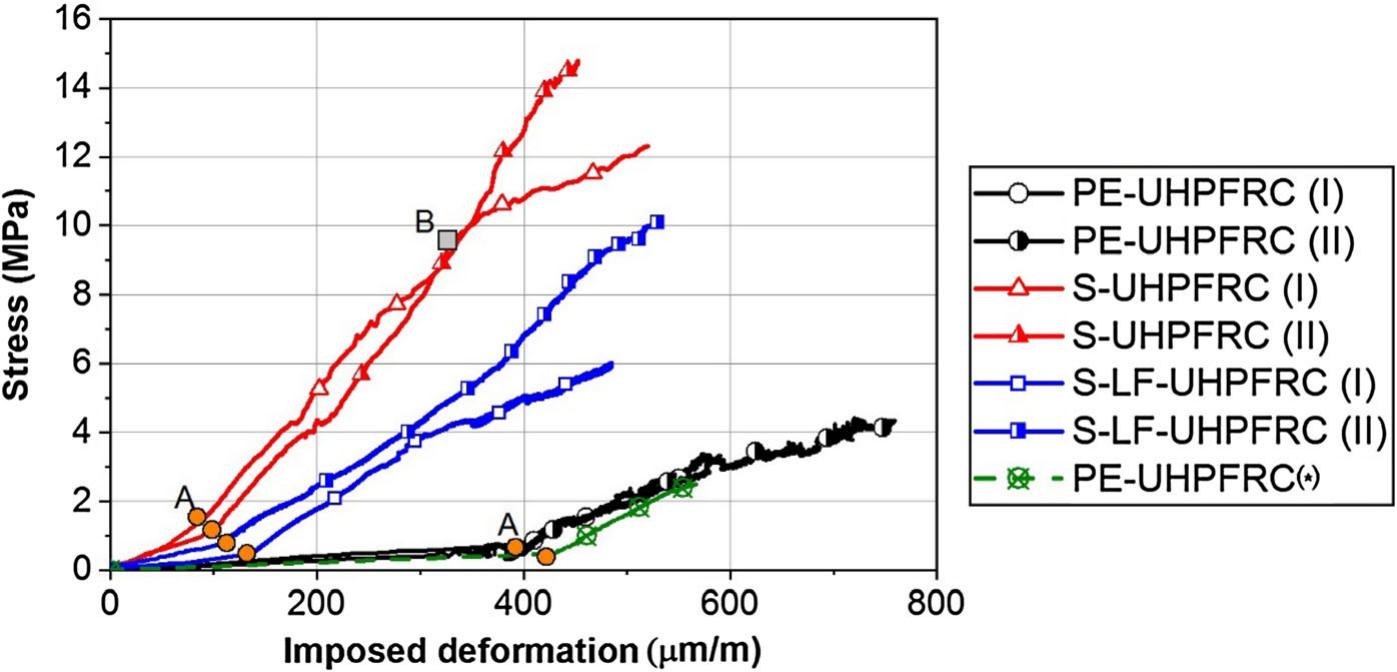
Tensile response of UHPFRC mixes under imposed shrinkage deformations

Figure 8 shows the tensile response of the UHPFRC mixes under imposed shrinkage deformations with very low loading rates. For the full restraint specimens, the free autogenous shrinkage deformation from the Free Setup was considered as the deformation acting on the specimen in Restraint Setup and causing the eigenstresses development. In case of the partial restraint specimen, PE-UHPFRC(*), 65% of the free autogenous shrinkage deformation from Free Setup, which was the real deformation acting on the specimen according to the degree of restraint, was used for the strain calculations.

All the mixes showed a bilinear tensile response (until the elastic limit) starting with smaller slope and continuing with a higher slope. Viscous effects (relaxation) significantly decrease the impact of the autogenous shrinkage on the development of eigenstresses, especially during the first linear part until point A (orange circles in Fig. 8). Considering the development of dynamic elastic modulus in Fig. 4, the first linear part of the tensile response with a lower slope finished when the dynamic elastic modulus reached 43 GPa in all the mixes. Table 4 shows the age and dynamic elastic modulus of the specimens at point A.

**Table 4:** Age and dynamic elastic modulus of the specimens at point A

	Age (h)	Dynamic *E* modulus (GPa)
S-UHPFRC	25	43
S-LF-UHPFRC	45	43
PE-UHPFRC	150	43

The second slope of the tensile response corresponded to the elastic modulus of the materials at a very low loading rate (that of the shrinkage deformations), which intrinsically includes the effect of relaxation due to the viscoelastic response. The average slopes of the second linear part were 37 GPa, 22 GPa, and 14 GPa for S-UHPFRC, S-LF-UHPFRC, and PE-UHPFRC, respectively. The tensile response of PE-UHPFRC(*) under partial restraint condition was almost superimposed on that for the PE-UHPFRC specimens under full restraint condition. This indicates that the tensile behavior of the material under imposed shrinkage deformation is independent of the degree of restraint; however, it takes longer for a partial restraint specimen to reach a given stress level than for a full restraint specimen. Furthermore, assuming that the deviation from linearity in the second slope indicates the beginning of the tensile hardening domain, it can be noticed that at point B in Fig. 8, the S-UHPFRC (I) entered the hardening domain when the eigenstresses in this specimen reached the elastic limit at 10 MPa which is lower than 12.3 MPa that stated in Table 2. The differences between these two values can be explained considering the fact that in this case, the specimen was loaded under very low loading rate of imposed shrinkage deformations, and the decrease in the strain rate considerably decreases the elastic limit of the UHPFRC [46]. This highlights the importance of considering eigenstresses in designing UHPFRC layers for rehabilitation.

## Conclusions

- The development of the elastic modulus started earlier in PE-UHPFRC compared with the other two mixes; however, the rate of development was higher for S-UHPFRC. The dynamic elastic modulus reached 45 GPa and 50 GPa for PE-UHPFRC and steel fiber mixes, respectively.
- The autogenous shrinkage was 2–3 times more in PE-UHPFRC compared to that in conventional UHPFRC with steel fibers, in the first days; however, this difference was reduced to 25% at 28 days. The combined effects of sand amount and steel fibers had a considerable effect on reducing the autogenous shrinkage.
- Replacing cement with limestone filler had a considerable effect on reducing the eigenstresses. Furthermore, using PE fibers instead of steel ones helped in reducing the eigenstresses. PE-UHPFRC showed 70% and 50% lower eigenstresses in the two tests under full restraint conditions compared with that in S-UHPFRC and S-LF-UHPFRC, respectively.
- The tensile response of UHPFRC mixes under imposed shrinkage deformations can be considered as bilinear, until the elastic limit. The first part with the smaller slope finished when the dynamic elastic modulus of the material reached 43 GPa. The tensile response of the material under shrinkage deformation was independent of the degree of restraint. The eigenstresses in one of the S-UHPFRC specimens passed the elastic limit and entered the hardening domain, which highlights the importance of considering eigenstresses in designing UHPFRC layers for rehabilitation.
